# Maximum entropy networks for large scale social network node analysis

**DOI:** 10.1007/s41109-022-00506-7

**Published:** 2022-09-28

**Authors:** Bart De Clerck, Luis E. C. Rocha, Filip Van Utterbeeck

**Affiliations:** 1 Department of Economics, Ghent University, Ghent, Belgium; 2 Department of Mathematics, Royal Military Academy, Brussels, Belgium; 3 Department of Physics and Astronomy, Ghent University, Ghent, Belgium

**Keywords:** Social networks, Maximum entropy networks, Disinformation identification, Network analysis

## Abstract

Recently proposed computational techniques allow the application of various maximum entropy network models at a larger scale. We focus on disinformation campaigns and apply different maximum entropy network models on the collection of datasets from the Twitter information operations report. For each dataset, we obtain additional Twitter data required to build an interaction network. We consider different interaction networks which we compare to an appropriate null model. The null model is used to identify statistically significant interactions. We validate our method and evaluate to what extent it is suited to identify communities of members of a disinformation campaign in a non-supervised way. We find that this method is suitable for larger social networks and allows to identify statistically significant interactions between users. Extracting the statistically significant interaction leads to the prevalence of users involved in a disinformation campaign being higher. We found that the use of different network models can provide different perceptions of the data and can lead to the identification of different meaningful patterns. We also test the robustness of the methods to illustrate the impact of missing data. Here we observe that sampling the correct data is of great importance to reconstruct an entire disinformation operation.

## Background

### Information spreading and opinion dynamics

Understanding the dynamics of opinions and information spreading on networks is key in our modern society where online social platforms allow everyone to voice their opinion and allow for rapid propagation of information. The spread of information on a social network can exhibit characteristics of complex contagions (Guilbeault et al. 2017). A broad overview of opinion dynamics can be found in Noorazar et al. (2020). Social media platforms use algorithmic personalisation to provide a customised experience to each user. In this personalised space, it has been found that algorithmic filtering might influence opinions in a social network (Perra and Rocha 2019). A user with malicious intent who manages to manipulate the platform or the algorithm so that he appears in the personalised space can nudge another user towards the desired opinion without the latter realising it.

### Disinformation campaigns

According to the definition of the European Commission, disinformation is ‘verifiably false or misleading information created, presented and disseminated for economic gain or to intentionally deceive the public’. Misinformation on the other hand is ‘verifiably false information that is spread without the intention to mislead, and often shared because the user believes it to be true’ (https://digital-strategy.ec.europa.eu/en/policies/online-disinformation). When disinformation is shared by a so-called ‘useful idiot’, it often turns into misinformation, because the person sharing it does not intend to mislead. Democratic elections in several countries have been plagued by both in recent years (Mazarr et al. 2019; Bradshaw and Howard 2018; Marchal et al. 2018; Woolley and Howard 2018; Linvill and Warren 2018). During the COVID-19 pandemic, there has been a flare-up of unreliable or low confidence information (Elhadad et al. 2021, 2020). Even though it is difficult to quantify the impact and the efficiency of such campaigns, the social media platforms and different national or supranational entities are actively encouraging research to detect, identify or attribute disinformation.

In this paper we focus on disinformation within the context of information operations: an active operation that is run by an actor with the intent to create desired effects on the will, the understanding and capability of its adversaries or potential adversaries in support of its own objectives. The analysis and identification of coordinated behaviour in social networks has previously been realised by using a user similarity network (Nizzoli et al. 2020) or by looking at account behaviour and activity patterns (Sharma et al. 2020; Pacheco et al. 2020), sometimes in a limited timeframe (Pacheco et al. 2020; Weber and Neumann 2021). Various tools and platforms exist and contribute to the fight against disinformation, e.g. Hoaxy (Shao et al. 2016) for the tracking of social news sharing, and various fact checking websites such as FactCheck (https://www.factcheck.org), PolitiFact (XXXX) or EUvsDisinfo (https://euvsdisinfo.eu). In addition, there are algorithms for identifying artificial accounts (Wang et al. 2019; Schuchard et al. 2019; Yang et al. 2020) and automatically classifying misinformation or propaganda (Guarino et al. 2020).

Currently only a few large social media platforms such as Reddit or Twitter give (sometimes limited) access to their data. On a regular basis, Twitter releases datasets that are suspected to be state-backed information operations (i.e. a disinformation campaign) via the Twitter information operations report (Twitter 2021). Twitter works in close collaboration with the Stanford Internet Observatory for an independent analysis (DiResta et al. 2020; Cryst and García-Camargo 2020; Bush 2020; Grossman et al. 2020) of the suspected accounts. In these case-by-case studies, both the content and the network topology are analysed. This always includes a subject matter expert who is able to understand the content of the messages and place it within the context of the targeted country.

Unlike other datasets e.g. concerning the 2016 elections in the United States, and despite their availability, the datasets from the Twitter information operations report have not been much studied. Some applications using these datasets include a semi-supervised ensemble-tree classifier model that was built to detect influential actors in a disinformation network (Smith et al. 2021) and the construction of coordination networks based on arbitrary behavioural traces shared among accounts (Pacheco et al. 2020). We use the datasets in the collection as a ground truth (curated by the social media platform) to detect disinformation operations.

### Specific applications on social networks

Despite the many applications such as link prediction (Parisi et al. 2018; Baltakiene et al. 2018), inferring network projections (Saracco et al. 2017), pattern detection (Squartini and Garlaschelli 2011) and network reconstruction (Mastrandrea et al. 2014) on all kinds of networks, the application of maximum entropy networks on social networks has been limited. This is mainly due to the scale of social networks and the associated computational cost. In some cases it is possible to circumvent this obstacle. For instance, when using the Directed Bipartite Configuration Model (DBiCM) (Reconstructing mesoscale 2019) on an interaction network generated from Twitter data, it is possible to determine the parameters without having to explicitly solve the system thanks to simplifications due to the choice of layers (Becatti et al. 2019).

The Bipartitte Configuration Model (BiCM) and the DBiCM are two models from the Exponential Random Graph Models (ERGM) (Park and Newman 2004; Hunter et al. 2012) family that have seen the largest amount of use cases on social media data, in part due to the computational aspect, but also because it allows the use of statistical tests to reduce the fully connected network to only the statistically significant connections. They have been used to identify significant user interactions in several applications such as identifying significant content spreaders (Caldarelli et al. 2021); observing that social bots play a central role in the exchange of significant content for political propaganda (Caldarelli et al. 2020); identifying significant content spreaders and identifying political alliances (Becatti et al. 2019); identifying significant interactions in Twitter disinformation datasets (De Clerck et al. 2022); analysing a semantic network during the COVID-19 pandemic (Mattei et al. 2021); characterising the behaviour of bots during the UK elections (Bruno et al. 2021).

### Contributions

This paper is an extended version of the work presented in (De Clerck et al. 2022), where only the DBiCM was used. At present we are able to consider additional network models thanks to the acceleration methods explained in the methods section. We analyse whether these advances make the methods suited for large scale social networks by applying them on interaction graphs from disinformation datasets and try to discover non-trivial patterns by comparing the observed interaction graphs with different null models from the ERGM family. The study is extensively extended by adding new results using other network models and by looking at the impact of node removal in the detection power. The contributions of this paper are the following: (1) we apply maximum entropy network models on the collection of datasets from the Twitter information operations report to identify statistically significant interactions between users. (2) We evaluate whether the recently proposed methods to solve a ERGM converge on large scale social networks. (3) We analyse whether the use of different members of the ERGM family can lead to the identification of interesting patterns and whether these results can be generalised. (4) We analyse the impact of disturbances in the data on the obtained results (sensitivity analysis).

## Data

The main data source is the Twitter information operations report which contains multiple datasets, linked to a specific disinformation campaign. Each dataset contains information about the users that were identified as connected to the disinformation campaign as well as all their tweets. We call these users ‘flagged users’. The flagged users can interact with other flagged users, but also with non-flagged users. The tweets with which flagged users interact that are authored by non-flagged users are not included in the dataset, but their tweet id is known. This allows these messages to be downloaded separately to identify which non-flagged users the flagged users interacted with. We refer to these messages as ‘external tweets’. Table 1 shows the percentage of messages that could be downloaded for each dataset in August 2021. It is worth mentioning that in some datasets, not all of the ‘flagged’ accounts actively tweeted. When this situation occurs, it leads to users who will never be connected, regardless of the network model used. Note that we did not include the latest datasets which were released in December 2021.

**Table 1:** External tweets for each dataset

Country	Period	${N_{RT}}$	${\%_{RT}}$	${N_{RP}}$	${\%_{RP}}$
Armenia	2021–2002	298	68.12	208	41.83
China	2020–2005	45,199	27.61	44,048	14.85
Cuba	2020–2010	1,225,007	76.98	104,601	62.31
Egypt	2020–2004	1,962,519	67.68	353,471	51.41
Honduras	2020–2004	222,281	76.28	101,606	57.19
Indonesia	2020–2002	367,482	44.66	536,776	36.09
Iran	2020–2010	213	66.67	620	50.16
Iran	2021–2002	74,070	79.46	79,106	67.37
Qatar	2020–2010	128,963	65.38	5496	48.65
Russia	2020–2005	306,980	72.59	449,637	59.53
Russia	2020–2010	197	80.20	72	90.28
Russia GRU	2021–2002	3237	64.20	2592	61.38
Russia IRA	2021–2002	20,509	90.17	4662	56.07
SA, EG and AE	2020–2004	7,045,922	53.85	774,943	51.22
Serbia	2020–2002	2,589,041	59.01	972,795	40.98
Thailand	2020–2010	5207	91.05	3814	87.05
Turkey	2020–2005	8,513,467	65.15	2,246,872	42.20

$N_{RT}, \%_{RT}$ and $N_{RP}, \%_{RP}$ denote the absolute number of messages and the percentages that could be downloaded for retweets and replies respectively

## Methods

We describe the different network representations we used, their associated null model, the network analysis methods and the methodology to evaluate the robustness of the methods.

### Network models

From the raw Twitter data we generate different networks which in turn can be compared with an appropriate null model.

#### User–user interaction network

We construct a weighted, directed user–user interaction network $G_{w}=(V,E)$. Each node corresponds to a user and each edge with weight $w_{ij}$ represents the amount of times user *j* interacts (retweets or replies) with user *i*. The direction of the edge matches the direction of the information flow. We consider each kind of interaction (retweet or reply) separately, because a reply does not necessarily entail support for the user being replied to. The third Twitter action, the quote, was not considered as the datasets contain limited or no relevant quotes.

#### Bipartite user-object interaction network

The user–user interaction network (weighted or not) is the most direct way of representing the interactions between users. We can also consider a (directed) bipartite network with two layers $\top$ and $\bot$. Note that we have not explicitly declared what the different layers of the (directed) bipartite network represent. Different layer choices are possible in function of the goal one wants to achieve:

1. Users and messages, where a directed edge from a user to a message indicates authorship and a directed edge from a message to a users indicates an interaction (e.g. a retweet) (Becatti et al. 2019). When this network is projected onto the user layer, we obtain the user–user interaction network $G_{w}$ that shows how information flows on the network.
2. Users and hashtags, where an edge between the two layers indicates that a users has interacted with a specific hashtag at least once. When this bipartite network is projected onto the hashtag layer, we obtain a semantic network (Mattei et al. 2021; Radicioni et al. 2020, 2021).
3. Verified and non-verified users, where an edge between the two layers indicates that a user has retweeted the other users at least once. When this bipartite network is projected onto the verified users layer, we obtain a network of discursive communities (Mattei et al. 2021; Bruno et al. 2021).
4. Users and an URL (or a domain), where an edge between the two layers indicates that a user has shared a specific URL (or linked to a domain) at least once. When this bipartite network is projected onto the user layer, we obtain a semantic network of external domains.
5. Less straight-forward options could be conceived as well e.g. hashtags and media content (image or video), political affiliation (if known) and hashtags, and so on.

We used the user-message and user-hashtag interaction networks. The advantage of using this bipartite network representation is that one can use a null model to filter out the noise, i.e. interactions between nodes that are not statistically significant.

### Community detection

In a network we can observe densely connected clusters of nodes that are poorly connected to each other. Such a cluster is called a network community. A widely used metric for community detection is modularity (Newman and Girvan 2004), which quantifies the quality of a particular division of a network in clusters. Formally, modularity is given by

$$\begin{aligned} {\mathcal{H}} = \frac{1}{2m}\sum _{c} \left( e_c - \gamma \frac{K_c^2}{2m} \right) \end{aligned}$$

where the sum goes over all communities *c* in the network, *m* is the total number of edges, $K_c$ is the sum of the degrees of the nodes in community *c* and $\gamma$ is a resolution parameter. Community structure can be detected by optimising the modularity over the possible divisions of a network (Newman 2006) i.e. we try to maximise the difference between the actual number of edges and the expected number of edges in a community. The Louvain method (Blondel et al. 2008) has been one of the go-to algorithms for community detection due to its speed and scalability. The Leiden algorithm (Traag et al. 2018) has been proposed as an improvement of the Louvain method because it is faster and guarantees well-connected communities. Additionally, it is capable of working with weighted, directed and multiplex networks. We use the Leiden method for community detection on different representations of the data to evaluate to what extent the representation is suited for the clustering of the users.

### Analytical maximum entropy framework

For the networks, we consider entropy-based null-models (Squartini and Garlaschelli 2011; Park and Newman 2004; Garlaschelli and Loffredo 2008). Given an observed network $G^*$, the maximum-entropy method consists of constructing an ensemble of networks ${\mathcal{G}}$ whose topology is random, apart from a controlled set of structural constraints, $\mathbf{C}$, measured on $G^*$. The ensemble is found by maximising the Shannon entropy *S*

$$\begin{aligned} S = - \sum _{G\in {\mathcal{G}}} P(G) \ln { P(G)} \end{aligned}$$

to obtain the least-biased ensemble. When the constraints are imposed on average on the ensemble, i.e. $\langle \mathbf{C} \rangle = \mathbf{C}$, this is called the canonical network ensemble (Bianconi 2018). This kind of ensemble is also known as an Exponential Random Graph (ERG) (Park and Newman 2004). The framework was extended with a fast and exact method for obtaining analytical results on the grand canonical ensemble in Squartini and Garlaschelli (2011). These random graph models can be used to find statistically significant discrepancies between a null model and a real network.

#### Scaling up

Estimating the parameters of an ERGM has a high computational cost as finding the parameters of a null model with local constraints requires solving at least ${\mathcal{O}}(N)$ non-linear, coupled equations (where *N* is the number of nodes in the network). One way of obtaining the parameters is using the equilibrium expectation algorithm (Byshkin et al. 2018). Under the hypothesis that the network is sparse, this method enabled estimating the parameters for a directed social network with over 1.6 million nodes (Stivala et al. 2020). Another method of reducing the complexity of the problem is to consider that identical constraints lead to the same value(s) of the hidden variables. This principle was explained in Garlaschelli and Loffredo (2008) and also used in Bie (2010) for the UBCM. In a lot of cases this allows to drastically reduce the size of the problem to the number of unique (tuples of) local constraints for other members of the ERGM family. In addition to this reduction, one might also use a gradient free fixed-point iterative scheme instead of a gradient based method (such as Newton’s method) to find a solution to the system of equations. This idea was put forward in Dianati (2016). The complexity reduction was integrated with the fixed-point approach in Vallarano et al. (2020). An extensive analysis of the performance of several numerical algorithms for solving different ERGMs (Vallarano et al. 2021) concluded that a fixed point recipe should be the preferred approach for large scale networks. This solves the issues of accuracy, speed and scalability and makes it possible to obtain the maximum likelihood parameters of large-scale networks for different members of the ERGM family.

### Null models

Two different null models were used for the BiCM and DBiCM network representations.

#### Bipartite configuration model

For a bipartite network $G_{BiP}$ with layers $\top$ and $\bot$ and with its biadjacency matrix *M* with entries $m_{i\alpha }$, the set of constraints $\mathbf{C}$ is composed of the degree sequences of the two layers $\top$ and $\bot$ of the network. This leads to a probability per graph that can be factorised as

$$\begin{aligned} P(M|{\boldsymbol{\gamma}},{\boldsymbol{\beta}}) = \prod _{i\in \top } \prod _{\alpha \in \bot } p_{i\alpha }^{m_{i\alpha }} \left( 1 - p_{i\alpha } \right) ^{1-m_{i\alpha }} \end{aligned}$$

where $p_{i\alpha } = \frac{e^{-\gamma _i-\beta _{\alpha }}}{1+e^{-\gamma _i-\beta _{\alpha }}}$ and ${\boldsymbol{\gamma}}$ and ${\boldsymbol{\beta}}$ are the $|\top |$- and $|\bot |$-dimensional Lagrange multipliers of the model (Saracco et al. 2017). The bipartite network can be projected onto one of the layers to obtain a mono-partite representation. The projection of the bipartite network onto one of the layers can be realised by using ${\mathcal{V}}$-motifs, a measure of similarity between two nodes on the same layer that considers the number of common neighbours. Consider $V_{ij}^*$, the observed number of ${\mathcal{V}}$-motifs between two nodes *i* and *j* on the $\top$ layer which can be written in function of the adjacency matrix as follows:

$$\begin{aligned} V_{ij}^* = \sum _{\alpha \in \bot } m^*_{i\alpha } m^*_{j\alpha } \end{aligned}$$

where $m_{i \alpha }^*$ denotes the observed value of $m_{i \alpha }$.

If ${\mathcal{V}}_{ij}^*$ exists, an edge exists between node *i* and node *j* in the projection. To reduce the amount of noise, one can use the fact that ${\mathcal{V}}_{ij}^*$ follows a Poisson–Binomial distribution (Wang 1993). The statistical significance of ${\mathcal{V}}_{ij}^*$ can be evaluated by its *p*-value:

1 $$\begin{aligned} p\text{-value}\left( {\mathcal{V}}_{ij}^* \right) = \sum _{{\mathcal{V}}_{ij} \ge {\mathcal{V}}_{ij}^*} f_{\text{PoissBin}} \left( {\mathcal{V}}_{ij} \right) \end{aligned}$$

In the above equation, $f_{\text{PoissBin}}$ denotes the probability distribution function of the Poisson–Binomial distribution with parameters $\mathbf{p} = (p_1,\ldots , p_\alpha , \ldots , p_{|\bot |})$, where $p_{\alpha } = \langle m_{i\alpha } \rangle \langle m_{j\alpha } \rangle$ and $\langle m_{i\alpha } \rangle$ denotes the expected value of $m_{i\alpha }$ under the null model.

#### Bipartite directed configuration model

For a directed bipartite network $G_{BiPD}$ with layers $\top$ and $\bot$ and with its biadjacency matrices *M* and $M'$ with entries $m_{i\alpha }$ and $m'_{\alpha i}$, the set of constraints $\mathbf{C}$ is composed of the directed degree sequences of the two layers $\top$ and $\bot$ of the network. This leads to a probability per graph that can be factorised as

$$\begin{aligned} P(M|{\boldsymbol{\gamma}},{\boldsymbol{\beta}},{\boldsymbol{\gamma}}',{\boldsymbol{\beta}}')&= \left[ \prod _{i\in \top } \prod _{\alpha \in \bot } p_{i\alpha }^{m_{i\alpha }} \left( 1 - p_{i\alpha } \right) ^{1-m_{i\alpha }} \right] \\ &\quad\cdot \left[ \prod _{i'\in \top } \prod _{\alpha ' \in \bot } {p'}_{\alpha 'i'}^{m'_{\alpha 'i'}} \left( 1 - {p'}_{\alpha 'i'} \right) ^{1-m'_{\alpha 'i'}} \right] \end{aligned}$$

where $p_{i\alpha } = \frac{e^{-\gamma _i-\beta _{\alpha }}}{1+e^{-\gamma _i-\beta _{\alpha }}}$, ${p'}_{\alpha 'i'} = \frac{e^{-{\gamma '}_{i'}-{\beta '}_{\alpha '}}}{1+e^{-{\gamma '}_{i'}-{\beta '}_{\alpha '}}}$, ${\boldsymbol{\gamma}}$ and ${\boldsymbol{\beta}}$ are the $|\top |$- and $|\bot |$-dimensional Lagrange multipliers of the model associated with the out-degrees and ${\boldsymbol{\gamma}}'$ and ${\boldsymbol{\beta}}'$ are the $|\top |$- and $|\bot |$-dimensional Lagrange multipliers of the model associated with the in-degrees (Baltakiene et al. 2018). Similar to the previous section, the directed bipartite network can also be projected onto one of the layers to obtain a mono-partite representation by using ${\mathcal{V}}$-motifs. Here the observed number of ${\mathcal{V}}$-motifs from node *i* to node *j* on the $\top$ layer can be written in function of the adjacency matrix as follows:

$$\begin{aligned} V_{ij}^* = \sum _{\alpha \in \bot } m^{*}_{i\alpha } m'^{*}_{\alpha j} \end{aligned}$$

where $m_{i \alpha }^*$ denotes the observed value of $m_{i \alpha }$. If ${\mathcal{V}}_{ij}^*$ exists, an edge exists from node *i* to node *j* in the projection. As before, the statistical significance of ${\mathcal{V}}_{ij}^*$ can be evaluated by its *p*-value:

2 $$\begin{aligned} p\text{-value}\left( {\mathcal{V}}_{ij}^* \right) = \sum _{{\mathcal{V}}_{ij} \ge {\mathcal{V}}_{ij}^*} f_{\text{PoissBin}} \left( {\mathcal{V}}_{ij} \right) \end{aligned}$$

In the above equation, $f_{\text{PoissBin}}$ denotes the probability distribution function of the Poisson–Binomial distribution with parameters $\mathbf{p} = (p_1,\ldots , p_\alpha , \ldots , p_{|\bot |})$, where $p_{\alpha } = \langle m_{i\alpha } \rangle \langle {m'}_{j\alpha } \rangle$ and $\langle m_{i\alpha } \rangle$ denotes the expected value of $m_{i\alpha }$ under the null model.

### Extracting the statistically significant network

Using the bipartite models described in the previous section, we can obtain a $|\top | \times |\top |$ matrix of *p*-values for the ${\mathcal{V}}$-motifs (cf. Eqs. 1 and 2). In the case of the BiCM this matrix will be symmetrical. Given the large amount of statistical tests to be executed, we use the Benjami–Hochberg procedure (Benjamini and Hochberg 1995) to control the false discovery rate at a fixed level $\alpha$ (set at 0.05 in our case). The procedure provides a limit value $p_{(k)}$ with which each *p*-value is compared. This limit value is obtained by sorting all *p*-values in ascending order and finding the largest value of *k* such that

$$\begin{aligned} p_{(k)} \le \frac{k}{m}\alpha \end{aligned}$$

where *k* denotes the sorted position and *m* denotes the total number of *p*-values to analyse. If the *p*-value of the observed ${\mathcal{V}}$-motif ${\mathcal{V}}_{ij}^{*}$ is less or equal than $p_{(k)}$, the link from node *i* to *j* is considered statistically significant and is maintained in the projection of the bipartite network onto the $\top$ layer.

### Robustness

It is not possible to collect all the data generated on Twitter (be it in real time or a posteriori), so a sample is generated by selecting a timeframe, a number of relevant keywords (which can be hashtags) and users. Messages from the selected users and messages containing the selected keywords end up in the dataset that will be used to generate interaction networks. From the network point of view, this amounts to sampling nodes and interactions. Sampling of (temporal) networks generally leads to biases (Achlioptas et al. 2006; Lee et al. 2006; Rocha et al. 2017).

Data collection on Twitter can be done using either the stream API or the search API. It is not unthinkable that, even when adapting the data collection query dynamically when using the stream API, one does not capture every message or interaction that might occur in a disinformation campaign while it is happening, as in general the modus operandi is dynamic and reactive. The above reasoning holds to some extent when working with the search API, as it is possible to use progressive insight to limit the amount of data that might have been overlooked. However another problem occurs when going back in time: as social media platforms are making more and more efforts to avoid disinformation on their platforms, specific posts might have been deleted or accounts might have been banned between the moment of their activity and the moment a researcher tries to obtain them. This can lead to missing parts of the relevant data as well. In order to evaluate the robustness, we consider the following approach: we gradually remove the hashtags with the highest degree in the bipartite user-hashtag graph and observe the number of nodes that becomes disconnected from the graph. We chose this approach over removing random nodes because scale-free networks are typically resilient with respect to random node removal. We also look at the impact of this routine when using a null model to filter out interactions.

## Results

We give an overview of the different results that were obtained. We start by providing some toy examples to illustrate the networks that can be obtained for different representations of the data and the effect the different methods have on community detection on and composition of those networks. We discuss one dataset of the Twitter information operations report in detail and provide an overview of the results for the other datasets.

### Toy examples

#### User-message interaction network

**Fig. 1:**
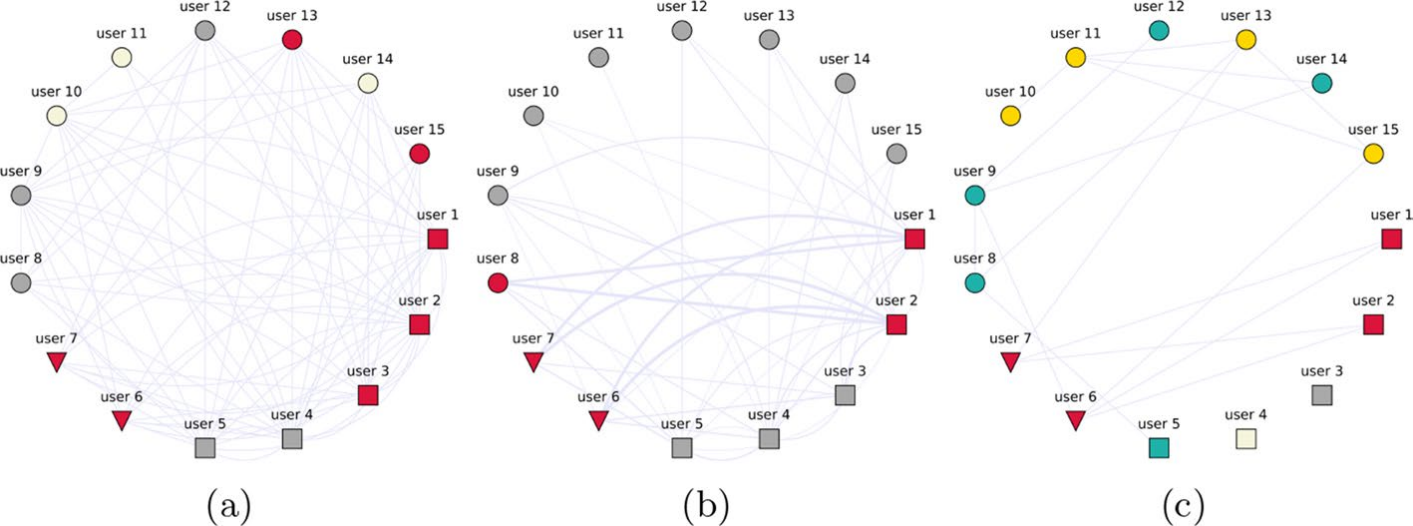
Toy example: community detection results for an interaction network based on users and messages sharing the same configuration: users 6 and 7 act as spreaders for users 1 and 2 in a synthetic network. Node shape represents behaviour (square: active poster, triangle: amplifier, circle: normal user). Node colour represents community membership. **a** Binary retweet network. **b** Weighted retweet network. **c** Filtered projected directed bipartite network

Consider the following setup for a small network of 15 users: users 1 through 5 author a large number of messages and have a certain visibility or reputation. Users 6 and 7 aim to artificially increase the visibility of users 1 and 2 by generating many interactions. The remaining users occasionally author a message and also interact among themselves in an organic way. We then use three different methods to generate an interaction network. First of all, we consider a binary network (i.e. omitting edge directionality and weight): if an interaction between users occurs, an edge will exist between them. Secondly, we consider a weighted version of the same network: the weight of the edge represents the amount of times a specific interaction occurred. Finally, we consider the bipartite users-message network projected onto the user layer where only the statistically significant links are maintained. We apply the Leiden algorithm for community detection on each of these networks. When using a binary network, information is lost and the identified communities give a distorted picture (Fig. 1a). Adding edge weight gives better results (Fig. 1b). Filtering out the statistically significant interactions using the DBiCM (Fig. 1c) identifies the ground truth even better. Note that in the case where users 1 and 2 author a large proportion of all messages and where users 6 and 7 also interact with a large portion of all other messages, their amplification effort would no longer be statistically significant. This can lead to disturbances in the community detection results.

#### User-hashtag interaction network

**Fig. 2:**
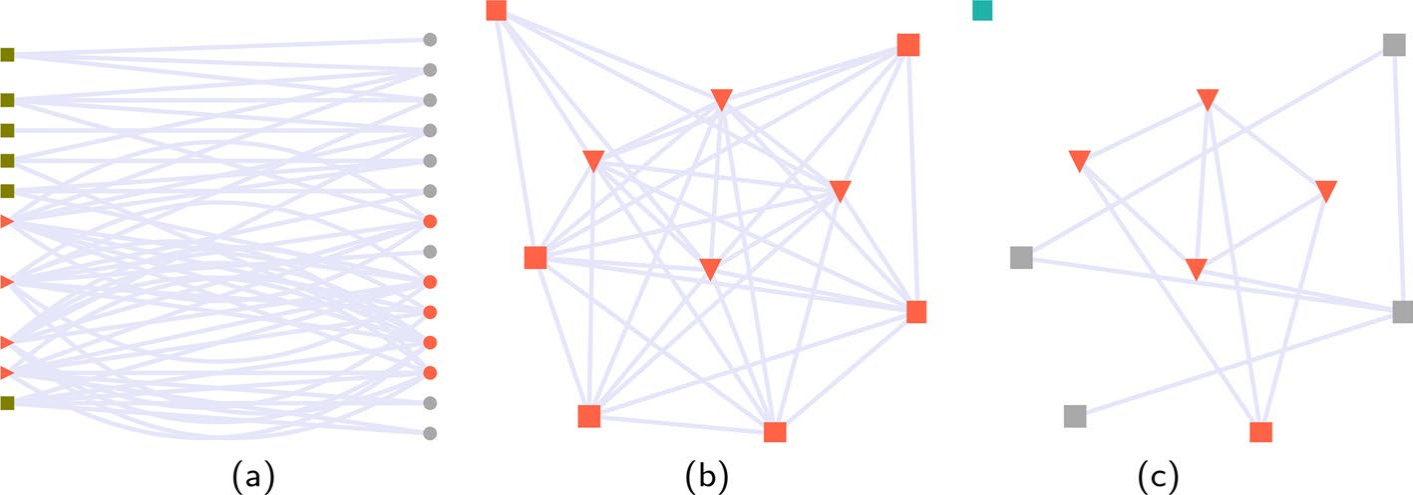
Toy example: results for an interaction network based on users and hashtags. **a** Bipartite network of users and hashtags. Gray squares: normal users, red triangles: flagged users, gray circles: hashtags, red circles: hashtags used mainly by flagged users **b** Projected bipartite network on the user layer. Squares: normal users, triangles: flagged users. Node colour represents community membership **c** Filtered projected bipartite network on the user layer. Squares: normal users, triangles: flagged users. Node colour represents community membership

Consider a bipartite user-hashtag network of 10 users with 15 hashtags, were four users are an organised group making use of a specific set of hashtags linked to their discourse. We generate the bipartite network, the projected network on the user layer and the projected network on the users layer with only the statistically significant links maintained (Fig. 2). After having obtained the filtered projection, we apply the Leiden algorithm for community detection. Using the communities discovered in the unfiltered network may be misleading because the normal users are confounded with the disinformation campaign. Filtering out the statistically significant interactions using the BiCM can lead to more accurate network communities.

### Validation

Because we want to evaluate to what extent the different models are able to reveal the underlying disinformation campaign we propose the following approach to evaluate the performance of a model: after the filtering has occurred, we consider every node that is still connected in resulting network to be a participant in the disinformation campaign. This is a rather harsh evaluation criterion, because there can (and most likely will) be non-flagged users still present in the projected network if they are the object of the disinformation campaign. Given that we are doing a binary classification, we use the Matthews correlation coefficient (MCC) $\Phi$ (Matthews 1975; Chicco and Jurman 2020) to quantify the performance. The MCC is defined in terms of the true positives (TP), false positives (FP), false negatives (FN) and true negatives (TN) as follows:

$$\begin{aligned} \Phi = \frac{TP \cdot TN - FP\cdot FN}{\sqrt{ (TP+FP)\cdot (TP+FN)\cdot (TN+FP)\cdot (TN+FN) }} \end{aligned}$$

Note that we define a flagged user as positive.

### Case study of the Honduras dataset

#### User-message interaction network

**Fig. 3:**
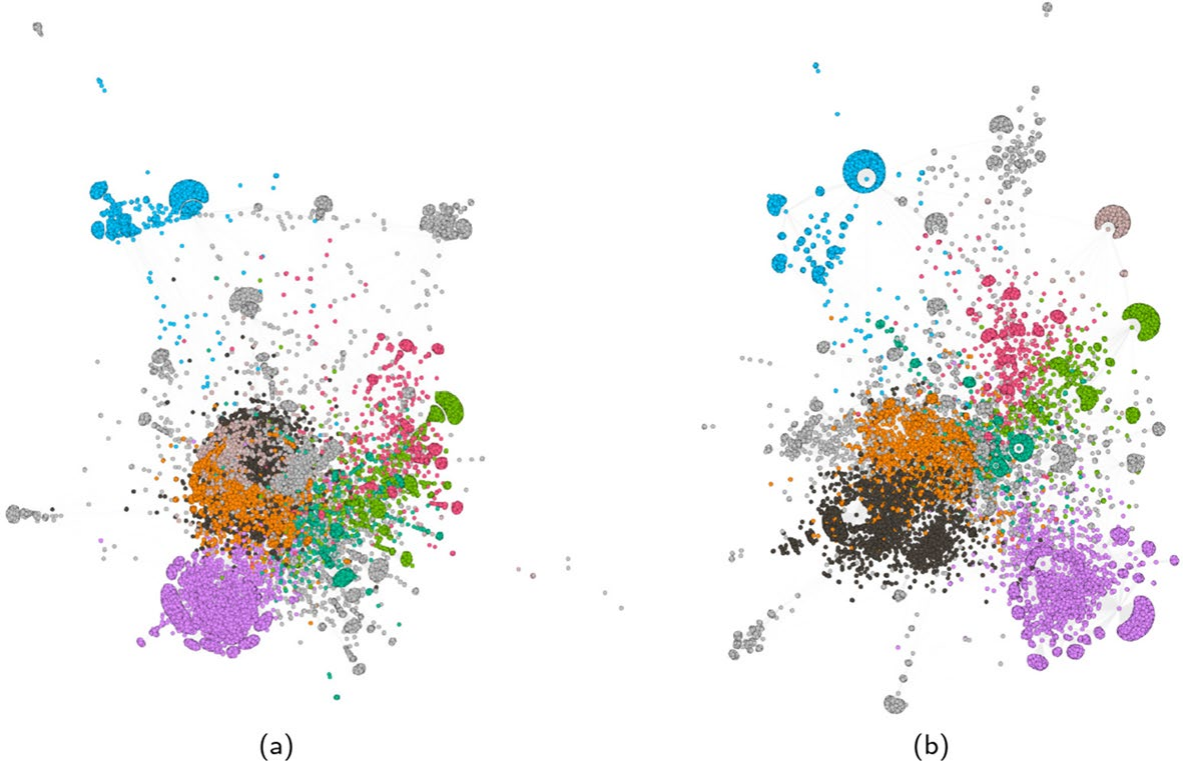
Honduras user-message bipartite interaction network projected on the user layer. Node colour indicates community membership. Node size is proportional to node degree. **a** Classic retweet network. **b** Projected retweet network

**Fig. 4:**
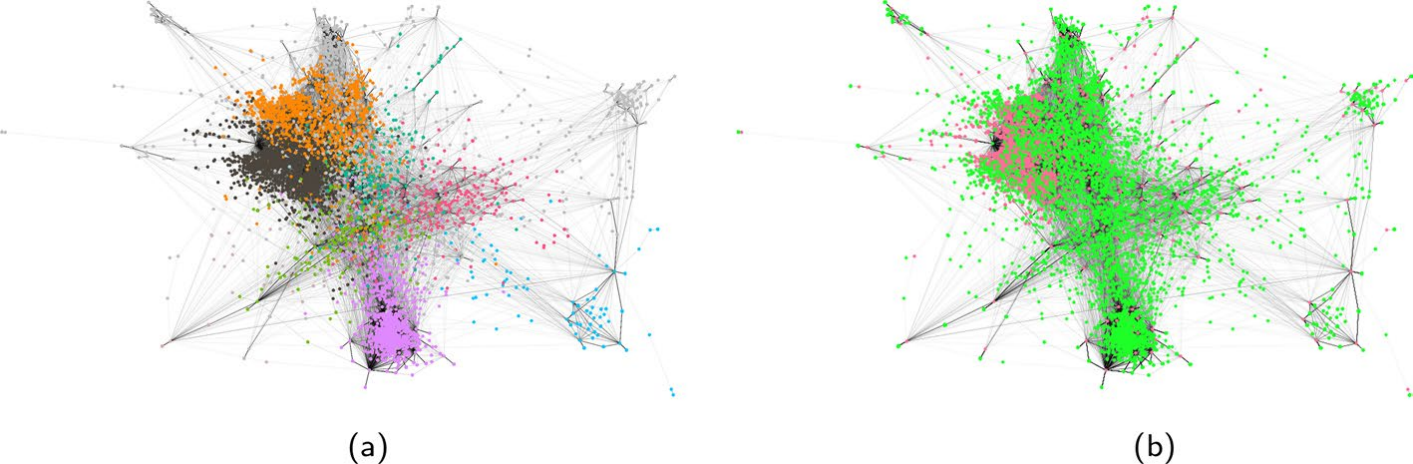
Largest component of the Honduras projected retweet user-message bipartite interaction network **a** Community membership. **b** Type of user (red: flagged, green: normal)

Figure 3 shows a sample for the retweet network of the Honduras dataset. Figure 4 shows the community detection result and the ground truth for the largest component of the projected user-message interaction network. When running community detection algorithms on both the retweet network and the projected user-message interaction network, we find that the communities and the central nodes found within them are in line with Cryst and García-Camargo (2020). Although not all edges from the ‘classical’ retweet network are maintained, the majority of flagged users are connected in the projected bipartite network. Because some edges were removed in the projection, the result of the community detection changed slightly, but without changing the overall conclusions on community composition. With a few exceptions, the removed edges are mainly low weight edges in the weighted network. In the Honduras network, the highest weight of a removed edge is 126 for an edge from @JuanOrlandoH to @tgaparicio. This particular situation where a ${\mathcal{V}}$-motif ${\mathcal{V}}_{ij}$ is considered non-significant, even with a high edge weight, can occur when a user *j* (here @tgaparicio) interacted with a large proportion of all the messages, while at the same time user *i* (here @JuanOrlandoH) authored a large portion of messages in the dataset. When looking at our performance criterion we find a $\Phi$ coefficient of 0.96 for the retweet graph and 0.8 for the replies graph.

#### User-hashtag interaction network

**Fig. 5:**
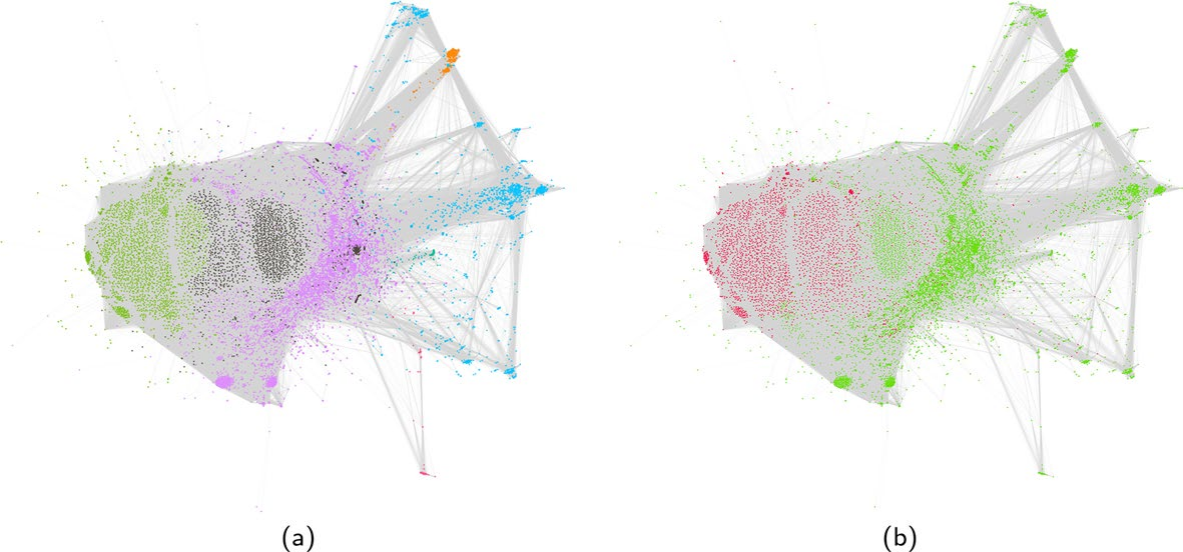
Largest component (94.9% of all nodes, 99.99% of all edges) of the non-filtered Honduras bipartite user-hashtag network projected on the user layer. **a** Community membership. **b** Type of users (red: flagged, green: normal)

**Fig. 6:**
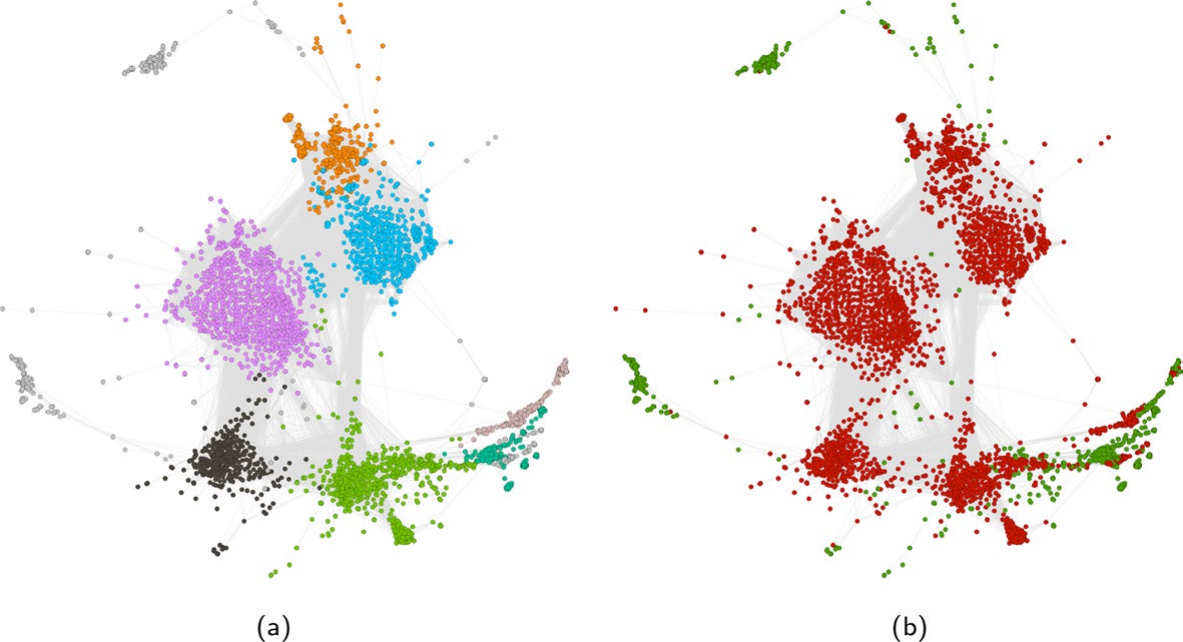
Largest component (20.5% of all nodes, 98.09% of all edges) of the filtered Honduras bipartite user-hashtag network projected on the user layer. **a** Community membership. **b** Type of users (red: flagged, green: normal)

Figures 5 and 6 show a sample for the filtered and non-filtered projection of the bipartite user-hashtag network on the user layer for the Honduras dataset. For both figures the results of community detection are shown on the left and the ground truth (flagged and non-flagged users) is shown on the right. Without filtering, there is a separation between flagged and non-flagged users, but there is more overlap within the communities (less pure). The network has 14,806 nodes and 4.2 million edges. The giant component accounts for 94.9% of all nodes and 99.99% of all edges. Most flagged users are present in the giant component. The flagged users who are not present are mainly users who did not author a message or used a hashtag, so they were never connected. After filtering, the network still has the same size, but the number of edges has been reduced to 256,000. The giant component now accounts for 20.5% of all nodes and 98.09% of the statistically significant edges. The percentage of flagged users in the giant component slightly decreased, but the ratio of flagged users in the giant component went up from 19.92 to 77.66%. When looking at our performance criterion we find a $\Phi$ coefficient of 0.63.

#### Robustness

**Fig. 7:**
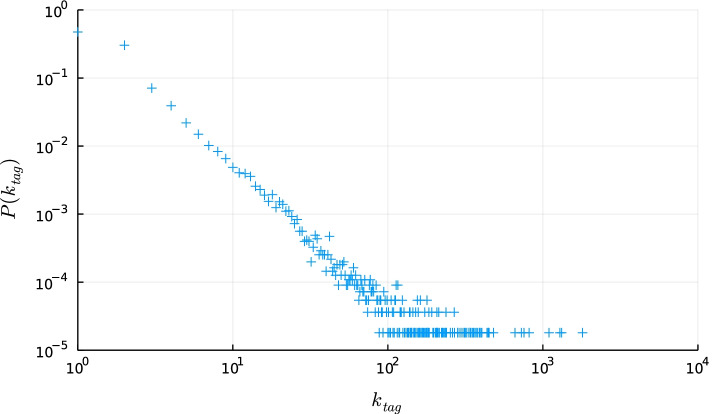
Degree distribution of the hashtags in the Honduras bipartite user-hashtag network

**Fig. 8:**
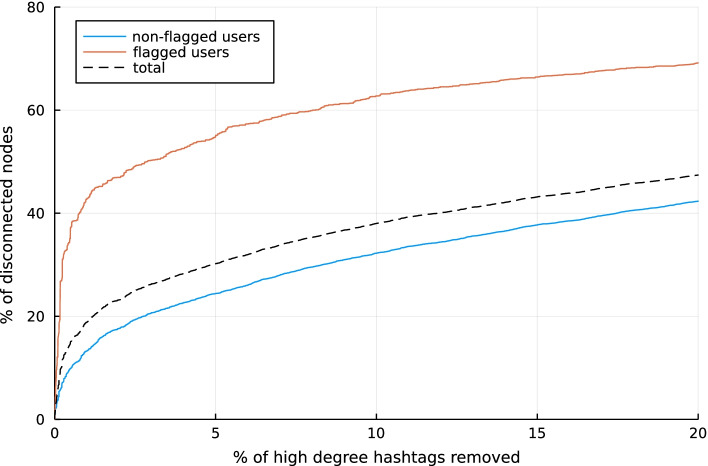
Impact of hashtag removal on disconnected users in the Honduras user-hashtag bipartite network

**Fig. 9:**
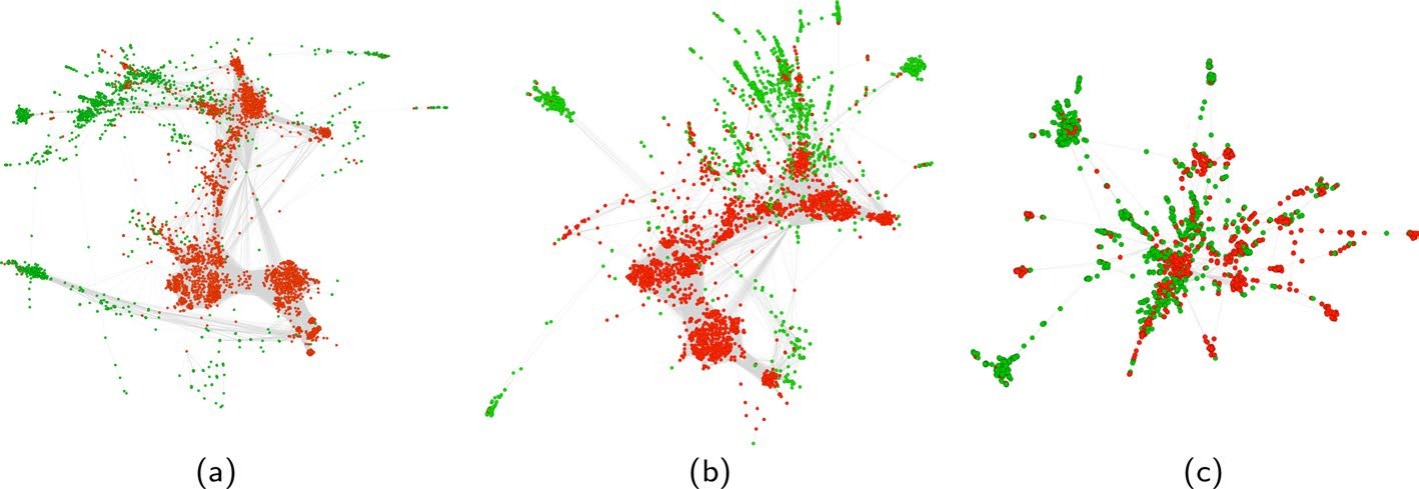
Evolution of the largest component in the Honduras user-hashtag bipartite network for successive removal of highest degree hashtags (note that the original largest component has a size of 3035 nodes). **a** 0.05% hashtag removal: 3486 nodes, 94% of all edges. **b** 0.1% hashtag removal: 3058 nodes, 91% of all edges. **c** 1% hashtag removal: 1535 nodes, 70% of all edges

The degree distribution of the hashtags in the bipartite users-hashtag network is highly skewed (Fig. 7). Figure 8 shows the effect of removing the most connected hashtags on the number of nodes that become disconnected. Even for a low percentage of hashtag removal, a considerable number of nodes becomes disconnected. This is in line with previous research where it was observed that participants in disinformation campaigns tend to use a more narrow or polarising discourse (Pacheco et al. 2020), which translates to a limited number of hashtags in this case. The effect of the node removal on the projection is shown in Fig. 9. As the percentage of removed hashtags increases, we can still observe a tight knitted structure in the largest component, but the amount of (flagged) nodes still present is reduced, as they are increasingly disconnected. At the same time the ratio between flagged and normal users decreases.

### General observations in Twitter information operations datasets

#### User-message interaction networks

The results for the other datasets are in line with the findings on the dataset described above: for both interaction types (retweets and replies), a large part of the edges of the projected user–user network are found to be statistically significant, with some exceptions such as the ‘*Russia (2020–2010)*’ and the ‘*Qatar (2020–2010)*’ datasets. Table 2 shows an overview of the results. We consider the method to fail when 1) both the percentage of matched edges in the retweet network ($\%_{M,RT}$) and the percentage of matched edges in the reply network ($\%_{M,RP}$) are less than 60% or 2) one of $\%_{M,RT}$ or $\%_{M,RP}$ is less than 30%. The apparent failure of the method on the dataset ‘*Russia (2020–2010)*’ is possibly due to the limited size of the network. The large drop in number of retained edges in the ‘*Qatar (2020–2010)*’ dataset is due to a single flagged user (@ShurafahAlthani) who interacted with more than 80% of all messages. A large portion of flagged users were connected to the disinformation network via this user and are disconnected in the projected network.

**Table 2:** Overview of matching edges between the weighted, directed and the projected bipartite network

Country	Period	${\%_{M,RT}}$	${\%_{M,RP}}$	N
Armenia	2021–2002	89.80	58.33	176
China	2020–2005	99.89	99.11	59,739
Cuba	2020–2010	55.01	80.10	172,073
Egypt	2020–2004	93.21	93.96	405,099
Honduras	2020–2004	83.90	82.83	51,816
Indonesia	2020–2002	65.93	86.47	33,142
Iran	2020–2010	75.19	47.77	197
Iran	2021–2002	89.48	88.11	15,555
Qatar	2020–2010	18.63	27.22	2764
Russia	2020–2005	80.85	93.18	61,938
Russia	2020–2010	6.67	6.15	9
Russia GRU	2021–2002	69.17	35.16	756
Russia IRA	2021–2002	34.18	77.41	1357
SA, EG and AE	2020–2004	84.23	97.41	945,842
Serbia	2020–2002	53.58	65.25	1,001,796
Thailand	2020–2010	72.32	61.35	2333
Turkey	2020–2005	81.82	90.86	1,069,045

$\%_{M,RT}$ denotes the number of matched edges for the retweet network. $\%_{M,RP}$ denotes the number of matched edges for the reply network. *N* denotes the number of nodes in the network

We found no correlation between the number of matching edges between the networks and (1) the percentage of external tweets that could be downloaded ($p=0.17$), (2) the number of external tweets ($p=0.42$) and (3) the number of nodes in the networks ($p=0.29$). Table 4 shows an overview of the performance metrics for the different datasets.

#### User-hashtag interaction networks

The results for the other datasets are in line with the results from the Honduras dataset, i.e. applying the BiCM and filtering out the statistically significant ${\mathcal{V}}$-motifs leads to a large component where the ratio of flagged users is significantly higher and in some cases is composed almost exclusively of flagged users. Table 3 shows an overview of several metrics for both the filtered and the non-filtered network. In general, the consequences of the filtering method are the following: the number of nodes situated in the largest component decreases and many nodes become disconnected; the majority of statistically significant edges is located in the largest component; the amount of flagged users in the largest component decreases (but to a lesser extent than the decrease of non-flagged users); the ratio of flagged users to non-flagged users in the largest component increases considerably. For two datasets (‘Iran (2020–2010)’ and ‘Russia (2020–2010)’) no statistically significant interactions were found. As was the case for previous model, this apparent failure may be due to the limited size of the networks. Table 4 shows an overview of the performance metrics for the different datasets. The values of the MCC are zero for the two datasets where no significant interactions were found. Another one that stands out is the ‘China’ dataset with a value close to zero, where a high value of false negatives leads to poor performance.

**Table 3:** Overview of the composition of the largest component for the user-hashtag network projected onto the user layer

		Non-filtered network				Filtered network			
Country	Period	$N_{C}$ (%)	$E_{C}$ (%)	$N_{C,f}$ (%)	$R_{C,f}$ (%)	$N_{C}$ (%)	$E_{C}$ (%)	$N_{C,f}$ (%)	$R_{C,f}$ (%)
Armenia	2021–2002	90.83	99.74	100.00	28.44	14.17	98.72	54.84	100.00
China	2020–2005	98.56	100.00	99.65	92.86	0.21	37.90	0.23	100.00
Cuba	2020–2010	97.28	100.00	99.80	1.33	11.94	99.32	87.60	9.52
Egypt	2020–2004	98.30	100.00	99.62	2.38	15.55	99.52	91.57	13.83
Honduras	2020–2004	94.90	99.99	99.86	19.92	20.50	98.09	84.09	77.66
Indonesia	2020–2002	98.00	100.00	99.69	2.30	0.77	77.49	32.51	95.89
Iran	2020–2010	42.78	87.64	77.67	100.00	N/A	N/A	N/A	N/A
Iran	2021–2002	93.60	99.97	99.50	1.55	6.00	83.24	67.66	16.44
Qatar	2020–2010	99.14	99.99	95.83	0.47	1.60	74.49	75.00	22.50
Russia	2020–2005	90.77	99.95	99.01	9.50	10.89	96.80	65.79	52.64
Russia	2020–2010	40.48	68.10	80.00	23.53	N/A	N/A	N/A	N/A
Russia GRU	2021–2002	88.53	99.87	95.56	5.80	1.67	84.00	24.44	78.57
Russia IRA	2021–2002	90.04	99.71	86.36	1.12	1.12	26.09	18.18	19.05
Serbia	2020–2002	97.27	100.00	99.87	18.42	25.61	99.49	94.60	66.25
Thailand	2020–2010	92.53	99.91	93.84	31.60	10.03	95.37	31.16	96.81
Turkey	2020–2005	98.62	100.00	99.47	4.23	31.39	99.91	84.70	11.31

$N_{C}$ denotes the percentage of all nodes that are present in the largest component. $E_{C}$ denotes the percentage of all edges that are present in the largest component. $N_{C,f}$ denotes the percentage of flagged users in the largest component with respect to the total number of flagged users. $R_{C,f}$ denotes the percentage of flagged users with respect to the total number of users in the largest component. A value “N/A” indicates that no significant interactions were found after filtering

**Table 4:** Overview of the performance of the different models using the MCC coefficient

Country	Period	$\Phi _{rt}$	$\Phi _{rp}$	$\Phi _{h}$
Armenia	2021–2002	0.81	0.59	0.63
China	2020–2005	0.41	0.26	0.03
Cuba	2020–2010	0.96	0.89	0.26
Egypt	2020–2004	0.88	0.51	0.29
Honduras	2020–2004	0.96	0.80	0.63
Indonesia	2020–2002	0.77	0.70	0.66
Iran	2020–2010	0.42	0.41	0.00
Iran	2021–2002	0.84	0.86	0.28
Qatar	2020–2010	0.85	0.72	0.29
Russia	2020–2005	0.89	0.83	0.49
Russia	2020–2010	0.63	0.77	0.00
Russia (GRU)	2021–2002	0.71	0.65	0.46
Russia (IRA)	2021–2002	0.82	0.74	0.22
Serbia	2020–2002	0.97	0.72	0.62
Thailand	2020–2010	0.47	0.11	0.49
Turkey	2020–2005	0.85	0.72	0.24

$\Phi _{rt}$ denotes the MCC coefficient based on the user-message retweet network. $\Phi _{rp}$ denotes the MCC coefficient based on user-message reply network. $\Phi _{h}$ denotes the MCC coefficient based on the user-hashtag network

## Discussion

### Network models

The parameters for both network models provide a more accurate view on the data and reveal traces of the underlying disinformation campaign. In function of the application and the social media platform under consideration, a suited network model should be selected. The bipartite user-message representation allows for a clearer overview of the user interactions whereas the bipartite user-hashtag representation reveals tight-knit discursive communities of the flagged users, in line with observations in previous research.

All methods used in the present work use a null model with a ‘simple’ interpretation of a network, i.e. a network formed exclusively by pairwise interactions. These networks were considered to be stand-alone and static in time. Possible extensions could include: (1) multilayer networks (Bianconi 2018), which allow to combine multiple representations. (2) temporal networks (Holme and Saramäki 2012), which allow to include the dynamics of the interactions. Methods to extract important links from temporal networks exist for techniques from visual temporal network analytics (Linhares et al. 2019), but testing is experimental (by user judgment), whereas within the maximum entropy statistical tests can be used. (3) higher-order networks (Bianconi 2021) and Exponential Random Simplicial Complexes (ERSC) (Zuev et al. 2015) to capture many-body interactions. Maximum entropy ensembles have been defined for these approaches (Courtney and Bianconi 2016; Bianconi 2013; Cimini et al. 2019). In principle, the methods used in this paper could be applied to these extensions. The speed of convergence and the usability at scale remains to be studied.

### Robustness and data collection

The analysis of the impact of removing some high degree nodes in the bipartite user-hashtag network showed that observing the disinformation network is highly sensitive to capturing the right hashtags. This should be taken into consideration by researchers who are monitoring social media streams or who want to compare new methods with earlier results. The datasets we used are curated by a social media platform, and show only a subset of the real-world interactions. Some hashtags used by a disinformation campaign could be used and shared by a broader public of non-flagged users, which would add additional nodes in the network. Whereas some specific hashtags might only be used by the members of a disinformation campaign. When these hashtags are not captured, it can be challenging to reconstruct the network and perform the analysis. For example, in a previous study, we showed that it was not possible to reconstruct a disinformation network when a large part of a dataset was removed by the social media platform (De Clerck et al. 2022).

The advantage of the datasets from the Twitter information operations report is that we have all the messages and interactions of the suspected users at our disposal. A possible downside is that we do not see them within their broader context, i.e. all the interactions of other users with the non-flagged users are missing from these datasets.

### Computational aspect

We found that the computational methods to obtain the maximum likelihood parameters proposed in Vallarano et al. (2021) scale well and are able to compute the parameters of the null model within seconds, even for the largest networks ($\approx$1M nodes) in the data collection. The costly step in the projection process is the computation of the *p*-values of the ${\mathcal{V}}$-motifs. The implementation in the NEMtropy package (Vallarano et al. 2021) computes all required *p*-values (using multithreading when available) and then proceeds with the Benjami–Hochberg procedure (Benjamini and Hochberg 1995). For large networks, we found that keeping all *p*-values in memory can be a bottleneck during the processing. For the largest networks in the collection some projections took almost 3 days to compute on an Intel i7-8700 with 64GB of RAM. This problem can be avoided by first computing and storing the *p*-values in chunks of a size determined by the size of the available memory and then using the fastSLU algorithm (Madar and Batista 2016), which requires linear time and does not require any *p*-value ordering.

An additional approach that could possibly accelerate the computation of the *p*-values is simulation: instead of computing the *p*-values of the Poisson–Binomial distribution, one could simply sample the network and compare the observed number of ${\mathcal{V}}$-motifs with a random sample fitted on a Poisson distribution as a simplification for the Poisson–Binomial distribution to estimate the *p*-value. Finally, in order to keep the size of the network limited when dealing with really large datasets, pruning (e.g. removing low degree nodes) before doing the computation might be another option, at the risk of losing useful information.

## Conclusion

We applied different maximum entropy network models on the collection of datasets from the Twitter information operations report to identify statistically significant interactions between users. The parameters for the models were computed using recently proposed methods and converged very fast for the different network sizes we considered. Using different members of the ERGM family can provide different views on the data. For the models used in this study, there is no evidence that one is better than the other because both are able to help reveal a disinformation campaign. Even though the overall scores in terms of our performance metric is higher for methods based on interactions, each method has its place, because it provides a different view on the data. We also found that capturing the right hashtags is of high importance if one wants to reconstruct the disinformation operation as a whole.

For all the figures related to the Honduras dataset the node layout was obtained using the ForceAtlas2 algorithm (Jacomy et al. 2014).

## Data Availability

The datasets generated and/or analysed during the current study are available in the Twitter Information Operations report (Twitter 2021). A Twitter developer account is required to access the datasets. A GitHub repository (https://github.com/B4rtDC/MaxEntSNA) is available with the script to extract the required external tweets from the datasets. Additionally, a Pluto notebook is provided to illustrate the process. Upon request, the authors can provide the tweet ids of the external tweets in alignment with the guidelines of the Twitter platform. The NEMtropy package (Vallarano et al. 2021) (https://github.com/nicoloval/NEMtropy) was used for the computation of the projections.

## Abbreviations

BiCM: Bipartite configuration model
DBiCM: Directed bipartite configuration model
ERG: Exponential random graph
ERGM: Exponential random graph models
ERSC: Exponential random simplicial complexes
MCC: Matthews correlation coefficient
UBCM: Undirected binary configuration model
